# Copy number variation of *CCL3L1* among three major ethnic groups in Malaysia

**DOI:** 10.1186/s12863-019-0803-3

**Published:** 2020-01-03

**Authors:** Jalilah Jamaluddin, Nur Khairina Mohd Khair, Shameni Devi Vinodamaney, Zulkefley Othman, Suhaili Abubakar

**Affiliations:** Department of Biomedical Science, Faculty of Medicine and Health Sciences, Universiti Putra Malaysia, UPM, 43400 Serdang, Selangor Malaysia

**Keywords:** Copy number variation, CNV, *CCL3L1*, Malaysian population, Paralogue ratio test, PRT

## Abstract

**Background:**

C-C motif Chemokine Ligand 3 Like 1 (*CCL3L1*) is a multiallelic copy number variable, which plays a crucial role in immunoregulatory and hosts defense through the production of macrophage inflammatory protein (MIP)-1α. Variable range of the *CCL3L1* copies from 0 to 14 copies have been documented in several different populations. However, there is still lack of report on the range of *CCL3L1* copy number exclusively among Malaysians who are a multi-ethnic population. Thus, this study aims to extensively examine the distribution of *CCL3L1* copy number in the three major populations from Malaysia namely Malay, Chinese and Indian. A diploid copy number of *CCL3L1* for 393 Malaysians (Malay = 178, Indian = 90, and Chinese = 125) was quantified using Paralogue Ratio Tests (PRTs) and then validated with microsatellites analysis.

**Results:**

To our knowledge, this is the first report on the *CCL3L1* copy number that has been attempted among Malaysians and the Chinese ethnic group exhibits a diverse pattern of *CCL3L1* distribution copy number from the Malay and Indian (*p* < 0.0001). The *CCL3L1* ranged from 0 to 8 copies for both the Malay and Indian ethnic groups while 0 to 10 copies for the Chinese ethnic. Consequently, the *CCL3L1* copy number among major ethnic groups in the Malaysian population is found to be significantly varied when compared to the European population (*p* < 0.0001). The mean/median reported for the Malay, Chinese, Indian, and European are 2.759/2.869, 3.453/3.290, 2.437/1.970 and 2.001/1.940 respectively.

**Conclusion:**

This study reveals the existence of genetic variation of *CCL3L1* in the Malaysian population, and suggests by examining genetic diversity on the ethnicity, and specific geographical region could help in reconstructing human evolutionary history and for the prediction of disease risk related to the *CCL3L1* copy number.

## Background

Malaysia is geographically located in Southeast Asia with a total land area of approximately 328,000 km^2^, separated by the South China Sea into two regions namely Peninsular Malaysia and East Malaysia (Labuan, Sabah, and Sarawak) [1]. The population of Malaysian is nearly 32 million which is equivalent to 0.42% of the world’s populations. Uniquely, Malaysia is populated by different ethnic groups which largely comprised Malay (68.8%), followed by Chinese (23.2%), Indian (7.0%), and other ethnic groups (1%) [2]. Delineation of ethnic groups in Malaysia based on genetic variation has been carried out on several genes such as *UGT1A1* and *CYP2*. UGT1A1*28 was found to be high in frequency among the Malay and Indian as compared to the Chinese ethnic group [3]. Furthermore, the presence of CYP2B6*6 among the Malay is also reported in a higher frequency compared to the other ethnic groups [4]. CYP2D6*10 is recently observed in both Malay and Chinese while CYP2D6*4 in the Indian ethnic group has been observed among breast cancer patients [5]. Besides, genome-wide of single nucleotide polymorphism (SNP) analysis also reported that the Malay group genetically differs from Chinese and Indian [6, 7]. This study suggested population-specific reference is important to explore unobserved common SNPs particularly for the Malay group which the data is limited in the database [6]. In addition, the gene with copy number variable is also less described among Malaysian, particularly in relation to the Malay who represent the largest populations of the Malay Archipelago- mainly present in Southeast Asia [8, 9].

Copy number variation (CNV) is a repetitive element in population diversity which results from the losses or gains of DNA segments of more than 1 kilobase which leads to different copies being presented [10, 11]. Among the reported genes with CNV, C Chemokine Ligand 3 Like 1 (*CCL3L1*) has been identified as one of the prominent genes with CNV in the current research field [12]. *CCL3L1* which was previously known as *LD78β*, is clustered on chromosome 17q12. This gene showed involvement in segmental duplication which was enriched with the immune response gene. According to the *Homo sapiens* Annotation Release 108, *CCL3L1* harboured at the base pairs of 250,577 to 252,466 on chromosome 17q21. The repeat unit of *CCL3L1* is approximately 90 kb in size and within the same repeat unit, lies together *CCL4L1*. Both *CCL3L1* and *CCL4L1* share 95% similarity with their own non-copy variable paralogues, *CCL3* and *CCL4,* respectively. Therefore, *CCL4L1* frequently acts as an additional parameter, allowing accurate determination of *CCL3L1* copy number [13–15]. *CCL3L1* encodes for LD78β, the isoforms of macrophage inflammatory protein 1α (MIP-1α) which only differs by three amino acids of the *CCL3* mature protein (LD78α) [16, 17]. The MIP-1α products play an important part in anti-tumour immunity and participate in the immunoregulatory and inflammatory processes [18, 19]. The truncated-2 form of LD78β produced by *CCL3L1* leads to high binding affinity to the CC chemokine receptor (CCR) 1 and 5 [16, 20]. This gene expression is in a different dosage which will eventually lead to the diversity of ethnic phenotypic and may contribute to ethnic-specific diseases [21, 22].

Interestingly, different ranges of *CCL3L1* diploid copy number have been observed among different populations such as 0 to 4 copies in the United Kingdom [23, 24], 0 to 11 copies in the United States of America [25], 0 to 14 copies in Africa [12, 26], 0 to 10 copies in China [27], 1 to 9 copies in Korea [28], and 0 to 10 copies in Japan [29]. While the other populations have been extensively sampled, Southeast Asian population have rarely been included. Although *CCL3L1* copy number has been proven to vary in different populations, such information is yet to be reported in Malaysia. Thus, this study aims to investigate the distribution of *CCL3L1* copy number among three major ethnicities in the Malaysian population. Indeed, the characterization of Malay genetic structure would be beneficial data in Southeast Asia which is poorly explored in genetic studies. Additionally, it may provide a starting base for fundamental insight into the association of *CCL3L1* with related diseases.

In order to report the copy number, there are varieties of platforms used such as real-time qPCR [12]. However, in this study, we adapted the Paralogue Ratio Test (PRT), a PCR-based technique which was designed specifically to simultaneously amplify two products with a set of primer; one which yields from the reference locus and another one from the test locus [24, 30]. PRT has shown a high accuracy level in measuring the copy number and the amount of DNA previously used was as little as 10 ng [24]. Furthermore, PRT is a system that offers a very simple, rapid, yet inexpensive method for quantifying copy number which is compatible with the application to genotype more than thousands of samples [30].

## Results

### Distribution of *CCL3L1* copy number among three major ethnics in Malaysia

**Fig. 1:**
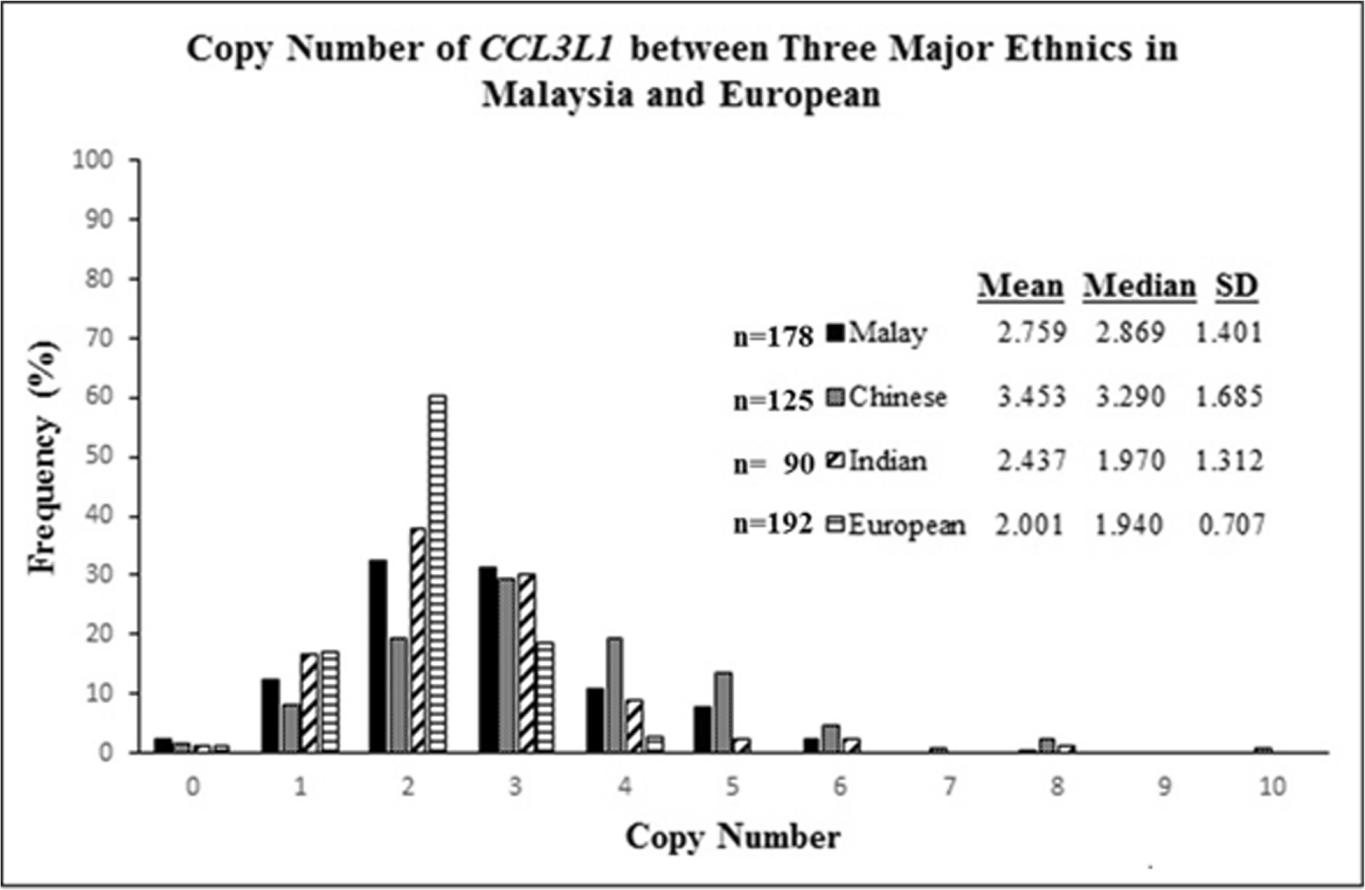
Distribution of *CCL3L1* copy number values among three major ethnic groups in Malaysia and Europe. A significant difference was observed in which the Malay and Indian showed a copy number range from 0 to 8, the Chinese copy number range was 0 to 10 while the Europeans have a copy number range of between 0 and 4

*CCL3L1* was found in variable copies where the Malay and Indian shared a similar range of 0 to 8 copies while the Chinese exhibit a wider range of 0 to 10 copies (Fig. 1). However, the Chinese (*n* = 125) and Malay (*n* = 178) shared a common copy number of 3 whereas a copy number of 2 was commonly found in Indian only (*n* = 90). The mean values were followed by the median values for each ethnic group comprising the Malay, Chinese and Indian are 2.759/2.869, 3.453/3.290 and 2.437/1.970 respectively. The histogram of the unrounded *CCL3L1* copy number obtained from major Malaysian ethnic groups was plotted to show the distribution of each group’s copy number (see Additional file 2). It is clearly showed that there was a grouping of data around each integer copy number even though there was no clear gap between each copy number’s division; rather, there was an overlap with each division at the end of the left and right sides for each copy number group. One-way ANOVA analysis revealed that the distribution of the *CCL3L1* copy number among those three major ethnic groups was significantly different with the *p-value* < 0.0001.

As we were emphasizing on the Malay CNV data as the major population, the Chinese and Indian ethnic groups were included for comparison purposes. Since the characteristics of the *CCL3L1* copy number among the Malay, Chinese and Indian groups have been established, the comparison to the European population was directly carried out. The *CCL3L1* copy number used for the European population in this comparison was obtained from a previous report by Walker et al. [24]. The distribution clearly showed a different pattern of *CCL3L1* copy number among the four groups (Fig. 1). The mean value of *CCL3L1* copy number reported for the European population were 2 copies. An analysis by the One-way ANOVA strongly showed a significant difference in the *CCL3L1* copy number between the three major ethnic groups in Malaysia when they were compared to the European population (*p*-value of < 0.0001). Table 1 showed the comparison of the copy number for three major ethnic groups in Malaysia and we had also included European, Norwegian and South African population as well. Result analysed by Χ^2^ showed significant difference of *CCL3L1* copy number range in Malaysian population between Malay and Chinese groups (Χ^2^ = 18.72; *p-*value = 0.0277). In addition, X^2^ analysis proved that the *CCL3L1* copy number distribution of Malay group is different when compared to European (*p*-value of < 0.0001), Norwegian (*p*-value of < 0.0001) and South African groups (*p*-value of < 0.0001) without consideration of the methodological approaches used.

**Table 1:** Comparison of the copy number followed by frequency (%) calculated in bracket between the ethnic groups in Malaysia and other reported populations

Copy number	Malaysia			European [24]	UK Biobank [31]	Norwegian [32]	South African [33]
	Malay	Chinese	Indian				
0	4 (2.25%)	2 (1.60%)	1 (1.11%)	2 (1.04%)	127 (2.55%)	11 (1.22%)	4 (0.81%)
1	22 (12.36%)	10 (8.00%)	15 (16.67%)	33 (17.19%)	1046 (21.00%)	159 (17.57%)	17 (3.46%)
2	58 (32.58%)	24 (19.20%)	34 (37.78%)	116 (60.42%)	2806 (56.33%)	527 (58.23%)	64 (13.0%)
3	56 (31.46%)	37 (29.60%)	27 (30.00%)	36 (18.75%)	853 (17.13%)	172 (19.01%)	131 (24.63%)
4	19 (10.67%)	24 (19.20%)	8 (8.89%)	5 (2.60%)	128 (2.57%)	35 (3.87%)	128 (26.02%)
5	14 (7.87%)	17 (13.60%)	2 (2.22%)	–	21 (0.42%)	1 (0.11%)	76 (15.45%)
6	4 (2.25%)	6 (4.80%)	2 (2.22%)	–	–	–	44 (8.94%)
7	–	1 (0.80%)	–	–	–	–	21 (4.27%)
8	1 (0.56%)	3 (2.40%)	1 (1.11%)	–	–	–	7 (1.42%)
9	–	–	–	–	–	–	–
10	–	1 (0.80%)	–	–	–	–	–
11	–	–	–	–	–	–	–
12	–	–	–	–	–	–	1 (0.20%)
Total	178	125	90	192	4981	905	493
X^2^	–	18.72	5.35	53.26	363.70	136.50	85.31
*P*-value	–	0.0277	0.6177	< 0.0001	< 0.0001	< 0.0001	< 0.0001

## Discussion

To the best of our knowledge, this is the first report on *CCL3L1* copies in Malaysia. In this study, we had investigated the distribution of the *CCL3L1* copies among the three major ethnic groups: Malay, Indian and Chinese using the PCR-based analysis (PRTs and microsatellites). Genetic diversity of the *CCL3L1* copy number among the Malaysian population is in agreement with the preceding findings [4, 6, 34]. The *CCL3L1* copy number range generally fall within the Asian regions which include Korea 1–9 copies [28], Japan 0–10 copies [29], China 0–10 copies [27], and India 0–7 copies [35]. According to a previous study, it was reported that the haplotype harbouring was more than 90% among the East Asia population (including Japan and Korea) could also be found in the population of Southeast Asia and South Asia including Malaysia and India [36]. However, further comparative analysis of the mentioned population could not be carried out due to insufficient data.

In the other regions, the *CCL3L1* copy number was reported in diverse ranges such as (0–4 copies) for Europeans [23, 24], (1–11 copies) for African American [25], (0–6 copies) for South American (Peruvian) [33], and (0–14 copies) for Africans [12, 26]. Thus, this data suggests that different population from different geographical regions has their own unique *CCL3L1* copy number range and that could act as a source of genetic diversity. The distribution of the *CCL3L1* copy number could be predominantly classified into three groups which include European, African and East Asian. This finding is in accordance with the recent report of the principal component analysis (PCA) plot on the global population. PCA has been widely performed to identify the genetic ancestry difference of individuals from various populations in order to generate the population structure [37]. These might strengthen the existence of different *CCL3L1* copy number between the populations. However, this should be further investigated to provide a strong conclusion on the relationship between *CCL3L1* diversity with different populations.

In addition, the significant difference of the *CCL3L1* copy number distribution among ethnicities found within the population (Malaysia) was in line with a study from the African population which suggested that ethnic background might influence the genetic variations [12, 38]. A particular reason behind copy number divergence between the populations and the ethnic groups remained unknown. Emphasizing the Malay in the Malaysian population, we noted a valuable finding when compared to other ethnic groups. The present result exhibited a different range of *CCL3L1* copy numbers between the Malay and Chinese groups in contrast to a previous study which reported that the Malay is genetically similar to the Chinese [6]. However, another study reported that *CCL3L1* is greater among Han-Chinese from Zhejiang province of China up to ten copies [27]. In addition the descendants of Chinese in Malaysia are predominantly from Southern China [39]. Therefore, extensive analysis including replicate or more studies is crucial in order to conclude the origin of Chinese in Malaysia. On the other hand, the analysed data displayed a sharing distribution of *CCL3L1* copy number between the Malay and Indian. Our analysis supports a study that revealed the presence of genetic influx from Indian to Malay as a result of admixture during the early existence of the Malay group [40]. This may provide a plausible explanation of the similar range of distribution of the copy number shared between these two groups as reported in this study.

Nevertheless, a whole genome sequencing study by Wong et al. (2013) stated that the Malay are significantly distinct from the Indian and Chinese [6, 34]. Intriguingly, it has been reported that Malay was a product of admixture between East Asian, South Asian, Austronesian, and Southeast Asian aboriginal people which lead to complex ancestry of Malaysian genetic data [34, 37, 41, 42]. Notably, the admixture event was also encountered within the subgroups of the Malay, observed among Malay in Peninsular Malaysia. A distinct genetic difference was reported between the Malay from the south and north of Peninsular Malaysia. This was probably due to the prolonged isolation followed by subsequent gene flow and adaptation, leading to changes in the genetic architectures of the Malay in the Malaysian population [34]. Therefore, a complex genetic diversity can be observed among the Malay in the Malaysian population. Unfortunately, genetic diversity data of the Malay and other ethnic groups in the Malaysian population is still insufficient to draw a concrete conclusion, particularly on the genes with CNV. This suggests that intensive investigations is needed in order to explore these ethnic groups further. Despite the fact that the single gene-focus in this study could not estimate the population’s history, it is a profoundly valuable contribution to the literature on *CCL3L1* copy number across the Malaysian population especially the Malay. Besides, the data is valuable in the efforts to investigate the genetic heterogeneity of *CCL3L1* among other ethnic groups in Malaysia and its association with related diseases.

Different types of platform or technique used for the determination of the *CCL3L1* copy number might influence the obtained range. In this study, a combination of PCR-based approaches includes PRTs and microsatellites were used although qPCR was frequently employed in the previous studies [12, 29, 43–45], and MLPA and ddPCR were recently used more [46, 47]. Therefore, the comparison between the copy number obtained between previous studies may not be as constant as it might generate more or less copy number than PRT. As for the PRTs assay, it has previously been shown to have a high degree of accuracy in predicting the integer copy number by identifying the discrete clusters with a consistency of microsatellite data [23, 24]. Even though there was a possibility of amplification on the pseudogene that might influence the final copy number reported, particularly for a high copy but the primers were specifically designed to amplify within intron 1, which was majority absent in pseudogene [22]. Meanwhile, it has been proven that qPCR tends to lead to false positive results as reported by Field et al. (2010) [48]. This is because qPCR primers amplify exon 3 within *CCL3L1,* and the pseudogene which resulted in extra copies of *CCL3L1* obtained [47]. Another finding also showed that qPCR might potentially lead to false-positive calls. Therefore, any studies of CNV association using only qPCR analysis have been suggested to perform a validation calling of the copy number [49, 50].

Until now, variable copies of the *CCL3L1* have yet to be investigated in the Asian populations compared to those from the European or American populations. Studies of *CCL3L1* copy number and its association with the susceptibility of related diseases have been found in European, Japanese, African, and Korean populations and a similar study could be conducted among Malaysians as well. Thus, more information about the CNV database particularly involving the Malay group is required as it represents one of the largest populations in Southeast Asia. The present finding had increased the CNV status data on the Malay along with the understanding of human genetic variations in relation to the different populations. It has also provided a fundamental implication in genetic studies. The impact of this study may offer a starting point for diseases susceptibility studies and other CNV diversity among the populations. The investigation of diseases susceptibility in association with *CCL3L1* variability in Malaysia is highly recommended.

Several limitations were identified in this present study. We clearly did not study on the association of *CCL3L1* copy number with any related diseases, thus we could not provide any association between those two. Besides, analysis on larger number of the sample size from each ethnic groups is highly suggested to increase the statistical power. This initiative might avoid any bias in mapping the genetic structure of Malaysian.

## Conclusion

In brief, this comprehensive study had allowed us to provide further insight into the distribution of *CCL3L1* copy number in the Malaysian population where a significant scattering has been ascertained among the three major ethnic groups. This present study offers a valuable genetic frequency in Malaysian population which currently underrepresented in population genetic studies. Future human evolutionary and association studies in relation to diseases will appreciate the availability of this data. Apart from that, ethnicity is another highly necessary variable for any research of genetic mapping.

## Methods

### Ethics approval, study design and subject recruitment

Ethical approval was obtained from the Ethics Committee of Universiti Putra Malaysia (UPM/TNCPI/RMC/1.4.18.1 (JKEUPM)/F2). All the subjects were given an explanation in regards to the study through the Respondent Information Sheet, and their written consent was obtained through the Respondent Consent Form. Whole blood samples were withdrawn in the range of 1 to 3 mL. A total of 430 unrelated volunteers comprising three major ethnics in Malaysia; Malay (*n* = 189), Chinese (*n* = 144) and Indian (*n* = 97) was successfully collected and quantified for the *CCL3L1* copy number. The subjects were students and staffs recruited from Universiti Putra Malaysia (UPM) Serdang, Selangor. Volunteers must fulfil the inclusion criteria as Malaysian, age between 18 and 55 years old, no mixed ethnicity for at least up to three (3) generations due to interracial marriage hence could be grouped as Malay, Chinese and Indian, and no clinical history of HIV or any autoimmune diseases. The determination of the ethnic groups was self-identified by the volunteers.

### DNA extraction

QIAamp DNA Mini Kit (QIAGEN, Germany) was used to extract the DNA from 200 μl of peripheral blood collected in BD Vacutainer® K2 EDTA tubes. Then, the concentration and purity of the extracted DNA were quantified using Nanodrop ND-1000 spectrophotometer (Thermo, USA).

### Quantification and validation of *CCL3L1* copy number

*CCL3L1* copies were first quantified using three Paralogue Ratio Tests (PRTs); CCL3A, CCL4A, and LTR61A, as described in the previous study [24]. In principle, PRT is a PCR-based method which only uses one pair of primer to simultaneously amplify two different loci, test (copy number variable) and reference (copy number invariable) [30]. The first system, CCL3A uses a set of primers designed to amplify both *CCL3L1* (test locus) and *CCL3* (reference locus) simultaneously on chromosome 17. The second system, CCL4A is developed to support the clarification of the *CCL3L1* copy number quantified by the CCL3A system where the primer for the CCL4A amplifies *CCL4L1* (test locus) and *CCL4* (reference locus). LTR61A is the third system, which also assists in verifying the copy number obtained which harbours on the long terminal repeat sequence located between *CCL3L1* and *CCL4L1* on chromosome 17 against the unlinked reference locus on chromosome 10 [24]. The three primer sets used to perform the capillary electrophoresis were fluorescently-labelled; HEX-CCL3A Forward (TCATAGTGGGTTCTCTGTTTC) with CCL3 Reverse (ATCCAGGGCTGCTTACTT), HEX-LTR61A Forward (AGTTTTCCTCTGCCTAGC) with LTR61A Reverse (TATTTATTTTAAGGTGTGCAC), and FAM-CCL4A Forward (GAGTCTGCTTCCAGTGCT) with CCL4A Reverse (GAGGAGTCCTGAGTATGGAG). The determination of the copy number was carried out by initially calculating the ratio obtained from the test to reference loci for each system.

To validate the integer of copy number calling by PRTs, two microsatellites lying within the *CCL3L1* region (TATC17 and TTAT17) were analysed in this study [24]. The amplification of the TATC17 region was carried out using non-labelled Forward primer TATC17 (CTTAGGGGGTCCTCTTGTC) and HEX-labelled Reverse primer TATC17 (CCAAAATCTGGATTAGTCAG). Meanwhile, for TTAT17, the amplification was carried out using FAM-labelled TTAT17 Forward primer (TCAGTTTTGCAAACGACCA) and non-labelled Reverse primer TTAT17 (GAAACTGGAAGGTGGAGATG). The amplified microsatellite products were resolved by capillary electrophoresis. The peak ratios of all the alleles to the smallest allele were calculated to infer the copy number, and the calculated total allelic ratio is considered as the integer copy number [24, 51].

### Quality control

European Collection of Authenticated Cell Cultures (ECACC) Human Random Control DNA samples known for their *CCL3L1* copy number from previous typing [24] were used as the internal control (reference) to infer the copy number for the unknown samples by deriving a linear regression equation from each PRT systems. The samples were C0075 (cn = 1), C0150 (cn = 2), C0007 (cn = 3) and C0877 (cn = 4). In addition, the typing missingness for the reference samples were also analysed to estimate the error rate of per-test. There was a total of 96 typing for all four (4) samples, and the incorrect calling copy numbers were 19 times, which mainly involved sample represented for three copy number (C0007). This analysis suggested that the error rates for the whole systems together were 20%, divided by three for three PRT systems (CCL3A, CCL4A and LTR61A) results in approximately 7% for each system. Armour et al. stated that in the determination of *DEFB* copy number, a single PRT test might contribute to approximately 8% of the error rate in determining the copy number [30]. Thus, the copy number typing of the reference samples from the whole set of experiments were considered as reproducible. Kolmogorov-Smirnov normality test was also performed on these samples in order to estimate the probability of the copy number group misclassification. All four copy number groups showed no significant difference, suggesting that the samples were normally distributed.

*CCL3L1* copy number for the unknown copy number samples showed 76% concordant either between two or three PRTs systems (*n* = 328). However, the agreement of the copy number calling between the PRTs system and microsatellite analyses found that (91%) 393 concordant out of 430 samples, leaving 37 samples being discordant. All the 37 samples with discordance were excluded from the statistical analysis due to the inconsistency of the copy number in all the assays (PRTs and microsatellites) (see Additional file 1). Thus, based on the agreement of the integer values between PRTs and microsatellites, only 393 samples were included for further statistical analysis.

### Statistical analyses

One-way ANOVA was used to calculate the significant difference of the *CCL3L1* copy number between three major ethnics as well as in comparison between the ethnic groups in Malaysia and the European population. Chi-square (Χ^2^) test of homogeneity was performed to identify the significance different in the frequency of *CCL3L1* copy number distribution among Malaysian as well as to the other populations. A statistical value is considered significant when the *p-value* is less than 0.05. The descriptive analyses between the population and the major ethnic groups in Malaysia were also calculated and described.

## Supplementary information

**Additional file 1.** Agreement between PRT and microsatellites. Samples with discordancy of copy number were excluded from further statistical analysis.

**Additional file 2.** Distribution of unrounded copy number among three major ethnics. Overlapping of copy number observed at the end (right and left sides) for each copy number group.

## Data Availability

The data sets of this article are included within the article and its additional files.

## Abbreviations

*CCL3L1*: Chemokine ligand 3-like 1
CNV: copy number variation
PRT: Paralogue ratio test
