# Supplementary figures and images for: Copy number variation of CCL3L1 among three major ethnic groups in Malaysia
Source: BMC Genet. 2020 Jan 3;21:1. doi: 10.1186/s12863-019-0803-3 (PMC6942282; doi:10.1186/s12863-019-0803-3)

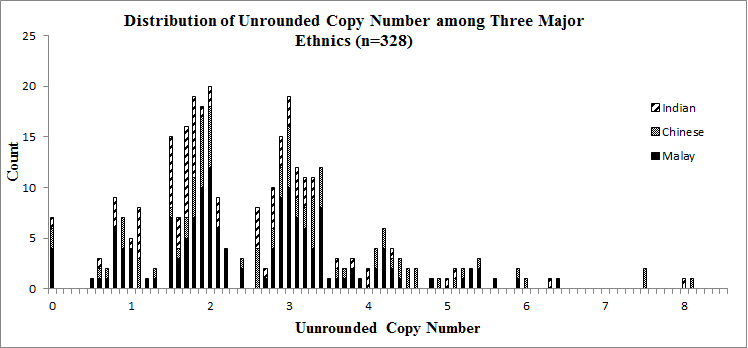

Supplement: Supplementary file 2 — Additional file 2. Distribution of unrounded copy number among three major ethnics. Overlapping of copy number observed at the end (right and left sides) for each copy number group. [file 12863_2019_803_MOESM2_ESM.tif]
